# The Immature Reticulocyte Fraction (IRF) in the Sysmex XN-1000V Analyzer Can Differentiate between Causes of Regenerative and Non-Regenerative Anemia in Dogs and Cats

**DOI:** 10.3390/ani14020349

**Published:** 2024-01-22

**Authors:** Alejandro Perez-Ecija, Carmen Martinez, Julio Fernandez-Castañer, Francisco J. Mendoza

**Affiliations:** 1 Department of Animal Medicine and Surgery, University of Cordoba, Campus Rabanales, Road Madrid-Cadiz km 396, 14104 Cordoba, Spain; v72mabem@uco.es (C.M.); fjmendoza@uco.es (F.J.M.); 2 Veterinary Teaching Hospital, University of Cordoba, Campus Rabanales, Road Madrid-Cadiz km 396, 14104 Cordoba, Spain; v72fercj@uco.es

**Keywords:** anemia, cats, dogs, hematology, immature reticulocyte fraction, reticulocytes

## Abstract

**Simple Summary:**

Anemia is a frequent problem in small animals with several underlying causes, each one with different treatments and prognosis. When facing a sick patient, veterinary clinicians need to correctly identify the problem behind this pathology as soon as possible. Some hematology analyzers provide a new parameter (the immature reticulocyte fraction, IRF) which has been used in human medicine for this purpose. Our results show that IRF is also able to differentiate between some causes of anemia. This new parameter may help veterinary clinicians to further advance in their diagnosis and focus on more specific tests for their patients.

**Abstract:**

The Sysmex XN-1000V analyzer can identify those reticulocytes with high RNA content and fluorescence, providing the immature reticulocyte fraction (IRF). While this parameter has been used in human medicine to identify the cause of anemia, few studies have focused on its use in veterinary medicine. In this study, we determined the IRF and related reticulocyte parameters in a large population of non-anemic and anemic dogs and cats (subclassified depending on the origin of their anemia). The IRF was significantly higher in hemolytic anemias compared to hemorrhagic ones in both species. Moreover, the IRF was significantly lower in dogs and cats with bone marrow failure than in other non-regenerative anemias and in both groups compared to pre-regenerative anemias. The accurate cut-off values for the differential in regenerative anemias and reference ranges for both species using the Sysmex XN-1000V are also reported. The measurement of the IRF in this analyzer can help clinicians to further classify the type of anemia in both species.

## 1. Introduction

Anemia is one of the most common laboratory abnormalities found in small animals [1]. The differential diagnosis of anemia usually requires patient history, clinical signs, biochemistry, and a serial assessment of multiple erythrocyte parameters. Anemia is usually classified as regenerative (RA) or non-regenerative (NRA) using the absolute reticulocyte count (RET) [2]. In turn, both RA and NRA can be subdivided according to the pathological mechanism (or cause) provoking the anemia (hemorrhage or hemolysis in RA; bone marrow failure or other causes in NRA).

The use of RET cannot differentiate between causes of anemia. Moreover, due to the delay in the reticulocyte release to the bloodstream, preregenerative anemias (PRA; acute hemorrhage) can be misdiagnosed as NRA [3].

Newer automated hematology analyzers, such as the Sysmex XN-1000V, determine the maturity level of circulating reticulocytes based on their RNA content and associated fluorescence and classify them as low- (LFR), medium- (MFR), and high-fluorescence reticulocytes (HFR). Low-fluorescence reticulocytes (LFR) are the most mature reticulocytes, with HFR being the most immature ones [4]. In addition, the Sysmex XN-1000V also calculates the immature reticulocyte fraction (IRF), defined as the sum of MFR and HFR percentages [3]. 

In human medicine, the IRF is recognized as an early marker of regeneration, increasing earlier than the RET in bone marrow engraftment studies or after chemotherapy [5]. Moreover, the IRF, either alone or together with the RET, is also useful in differentiating between the causes of NRA and RA in humans [6,7]. Fewer studies have focused on HFR (showing similar applications to the IRF), while the clinical value of MFR and LFR is not clear at the moment [8]. 

While the IRF has been studied in healthy and anemic dogs, no previous report has compared this parameter between both groups. In anemic dogs, the IRF shows higher values in RA compared to NRA [9] and is able to differentiate between PRA and other types of NRA [3]. However, its utility to classify anemia based on etiologic causes has yet to be elucidated. Moreover, to the authors’ knowledge, the IRF has also not yet been evaluated in anemic cats. Finally, the reference intervals (Ris) of these reticulocyte subpopulations have not been reported using the Sysmex XN-1000V in small animals. 

Therefore, the objectives of this study were the following: (a) to compare the IRF values between healthy and anemic dogs and cats using the Sysmex XN-1000V; (b) to determine the utility of the IRF in the differential diagnosis of canine and feline anemias using the Sysmex XN-1000V; and (c) to characterize the reference intervals (RIs) of these parameters using the Sysmex XN-1000V and evaluate the effect of age and sex in healthy dogs and cats. 

## 2. Materials and Methods

### 2.1. Case Selection and Classification

Animals were retrospectively selected from those referred to the Veterinary Teaching Hospital of the University of Cordoba between January 2019 and May 2023 with complete medical records and a CBC analysis using the XN-1000V analyzer (Sysmex Corporation, Kobe, Japan). 

**Figure 1:**
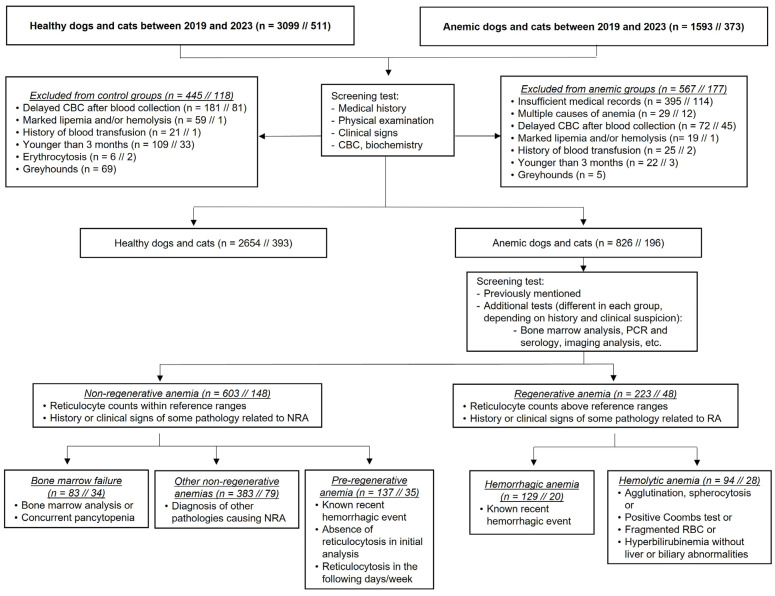
Flow diagram of case enrollment in the study.

Dogs and cats were divided (Figure 1) into control (healthy non-anemic patients sampled during their annual health program check) and anemic animals. Animals were considered healthy based on a normal clinical history and physical examination, and hematology results within reference ranges in our institution: red blood cell count (RBC) 5–8.6 × 10^6^/μL, hematocrit (HCT) 35–52%, hemoglobin (Hb) 12–20 g/dL, platelet count (PLT) 200–500 × 10^3^/μL; and RBC 5–12 × 10^6^/μL, HCT 30–45%, Hb 10–15 g/dL, PLT 250–550 × 10^3^/μL, for dogs and cats, respectively. Anemia was diagnosed when the RBC, HCT, and Hb (all of them) were below the reference ranges (Figure 1). The absolute reticulocyte count (RET) was used to differentiate between RA (RET > 110 × 10^3^/μL and >50 × 10^3^/μL in dogs and cats, respectively) and NRA (RET below those levels). Animals with RA and NRA were further subclassified according to the etiologic cause of anemia (Figure 1) in the following groups: hemorrhagic anemia (HEM) and hemolytic anemia (LYS) in RA; bone marrow failure (BMF), other non-regenerative anemias (OTH, inflammatory disease, endocrinopathy, chronic renal failure, etc.), and preregenerative anemias (PRA) in NRA. In order to classify these animals, their history, clinical signs, and serum biochemistry results were used. Additional results from other diagnostic tests (bone marrow examination, blood PCR for infectious diseases, thoracic radiographs, abdominal ultrasound, etc.) were also used for further classification into specific subgroups (Figure 1). Animals with PRA were recognized using serial CBC analyses as previously described [3]. Due to economic restraints and owners’ preferences, not all the listed diagnostic tests were performed in every case. Nonetheless, only cases with sufficient clinical information and diagnostic results to allow a definitive classification in one of the aforementioned groups were included in the study. 

The exclusion criteria were the following (Figure 1): delayed CBC analysis after blood collection, lack of a definitive diagnosis, history of blood transfusion within 30 days before sampling, animals younger than 3 months old (to avoid unrecognized hematological abnormalities due to age-related changes) [10], and Greyhounds (due to specific breed-related ranges) [11]. No repeated analyses from the same patient were included and only the initial CBC was considered.

The Institutional Animal Care and Use Committee of the Veterinary Teaching Hospital of the University of Cordoba (2021PI/02, approval date: 8 February 2021) approved this study and owner consent was obtained.

### 2.2. Sampling and Measurements

Blood samples were collected by cephalic or jugular venipuncture in an EDTA-containing tube and analyzed using a Sysmex XN-1000V analyzer (Sysmex Corporation, Kobe, Japan). The data from samples not measured within 30 min were discarded. The instrument’s graphical information was inspected and the following parameters were retrieved: RBC, HCT, Hb, mean cell volume (MCV), mean corpuscular hemoglobin (MCH), mean corpuscular hemoglobin concentration (MCHC), red cell distribution width (RDW), RET (named as RET# in the Sysmex XN-1000V), LFR, MFR, HFR, and IRF.

The samples with lipemia or hemolysis were excluded. An internal quality assessment was performed weekly using two levels of the manufacturer’s quality control material (XNCHECK Level 1 and Level 2; Sysmex Corporation, Kobe, Japan).

### 2.3. Statistical Analysis

Normality was assessed by a Kolmogorov–Smirnov test. The results were expressed as the mean ± standard deviation (SD) or median and interquartile range (IQR, 25th–75th percentiles) as appropriate. The median and percentiles were calculated using Tukey’s Hinges test. 

Groups were compared using the Kruskal–Wallis test with Dunn’s post hoc test or one-way ANOVA with Tukey’ post hoc test depending on normality (unpaired *t*-test or the Mann–Whitney test, according to normality, when only two groups were compared). Spearman’s or Pearson’s coefficients were used to determine the correlations between parameters as appropriate. 

Receiver operating characteristic (ROC) curve analysis was performed to determine the sensitivity, specificity, and cut-off values of the reticulocyte parameters in the differential diagnosis of each group. The accuracy for this differential was evaluated based on the area under the curve (AUC), according to previously published guidelines [12]. In short, accuracy was classified as excellent with an AUC between 0.9 and 1.0; good (0.8–0.9); fair (0.7–0.8); poor (0.6–0.7); and fail (<0.6).

Following recommendations from the American Society of Veterinary Clinical Pathology, reference intervals were obtained with a robust or nonparametric method as appropriate, using a dedicated software (Reference Value Advisor v. 2.1. freeware. Available at: http://www.biostat.envt.fr/reference-value-advisor/. Accessed on 27 July 2023), and are presented as two-sided 90% confidence intervals [13]. Only data from the control groups were used to calculate the reference ranges.

For the second objective, animals were grouped based on sex and age (<1 year old; 1–10 years old; >10 years old). 

Statistical analyses were performed using two statistical softwares (GraphPad Prism 9, San Diego, CA, USA and R software version 4.3.2), and values with *p* < 0.05 were considered significant.

## 3. Results

A total number of 3099 dogs and 511 cats were retrospectively enrolled into the healthy group (Figure 1). Four hundred and forty-five dogs and one hundred and eighteen cats were excluded following our criteria; thus, a total of two thousand six hundred and fifty-four dogs (*n* = 2654) and three hundred and ninety-three cats (*n* = 393) were finally included in the study.

On the other hand, a total number of 826 (out of 1593) dogs and 196 (out of 373) cats were recognized as anemic, with 603 dogs and 148 cats included in the NRA group and 223 dogs and 48 cats in the RA group (Figure 1). In the NRA groups, 83 dogs were subclassified as BMF, 383 as OTH, and 137 as PRA; while 34 cats were included in BMF, 79 cats were classified as OTH, and 35 cats as PRA. Concerning RA, a total of 129 dogs and 20 cats were included in the HEM groups, with 94 dogs and 28 cats classified in the LYS groups (Figure 1). 

### 3.1. Reticulocyte and Erythrocytic Parameters in Healthy vs. Anemic Animals

Healthy dogs and cats showed significantly different (*p* < 0.05) RET than patients with NRA or RA (Table 1). In healthy animals, the IRF was significantly lower (*p* < 0.02) compared to both NRA and RA patients. 

### 3.2. Reticulocyte and Erythrocytic Parameters in NRA vs. RA Animals

When both types of anemia were compared, the RET and IRF were significantly lower (*p* < 0.05) in NRA compared to RA in both species (Table 1). 

### 3.3. Reticulocyte and Erythrocytic Parameters in Animals with Different Causes of NRA

**Figure 2:**
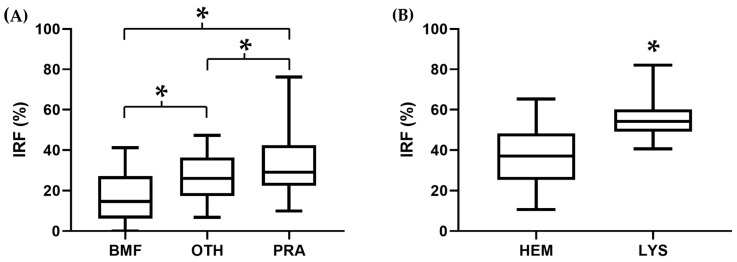
Immature reticulocyte fraction (IRF) in different causes of canine (**A**) non-regenerative and (**B**) regenerative anemia. Box and whisker plot. The box represents the first and third quartiles, the middle line represents the median, and the whiskers represent the 5–95% range. * *p* < 0.05 vs. the other group.

**Figure 3:**
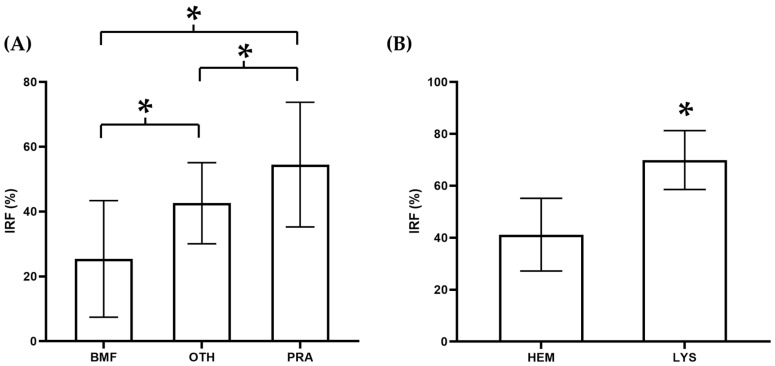
Immature reticulocyte fraction (IRF) in different causes of feline (**A**) non-regenerative and (**B**) regenerative anemia. Box represents the mean value and bar the standard deviation. * *p* < 0.05 vs. the other group.

No differences were observed in the RET between groups, with the only exception of dogs with BMF, which showed significantly lower (*p* < 0.05) RET compared to other NRA. The IRF was significantly different (*p* < 0.009) between every group in both species (Table 2 and Figure 2A and Figure 3A). 

### 3.4. Reticulocyte and Erythrocytic Parameters in Animals with different Causes of RA

No differences were observed in the RET between animals with hemolytic and hemorrhagic anemia. In both species, the IRF was significantly higher (*p* < 0.0001) in LYS compared to HEM (Table 3 and Figure 2B and Figure 3B).

### 3.5. ROC Curves for the Differential Diagnosis of Anemia in Dogs and Cats

**Figure 4:**
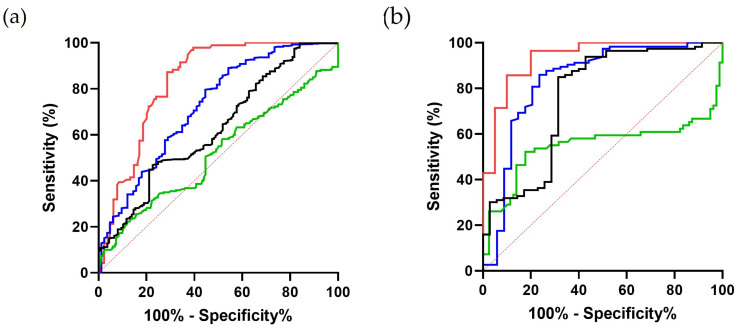
Receiver operating characteristic curve of IRF: (**a**) canine anemia (HEM vs. LYS: red line; BMF vs. other NRA: blue line; OTH vs. other NRA: green line; PRA vs. other NRA: black line); (**b**) feline anemia (HEM vs. LYS: red line; BMF vs. other NRA: blue line; OTH vs. other NRA: green line; PRA vs. other NRA: black line). Dotted red line: line of no-discrimination or random classifier.

In animals with RA, the IRF (%) showed an excellent accuracy in cats and a good accuracy in dogs for differentiating between HEM and LYS (Table 4 and Figure 4). In NRA, the IRF was good (cats) and fair (dogs) to differentiate BMF from other causes of NRA; and fair (cats) and poor (dogs) to differentiate PRA from other NRA. The IRF failed to identify OTH (Table 4 and Figure 4). 

### 3.6. Reference Intervals for Reticulocyte Subpopulations in Healthy Dogs and Cats

The reference intervals for each reticulocyte parameter in healthy dogs and cats are shown in Table 5. The frequency distributions for the IRF in both species are compiled in Supplementary Figures S1 and S2. 

### 3.7. Correlations with Other Parameters and the Effect of Age and Sex in Reticulocyte Subpopulations in Healthy Dogs and Cats

No strong correlations were observed between any reticulocyte subpopulation and the main red blood variables (HCT, RBC, HGB, RET), neither in dogs nor in cats (Supplementary Table S1). However, the IRF was significantly (*p* < 0.05) correlated with the rest of the subpopulations: LFR (*r* = −1.00, in both species), MFR (*r* = 0.68 in dogs; *r* = 0.27 in cats), and HFR (*r* = 0.95, in both species) (Supplementary Table S1). 

Younger cats (<5 years old) showed a significantly (*p* < 0.0001) higher LFR and lower IRF and HFR compared to cats above 10 years old. No other differences were observed between other age groups or in dogs (Supplementary Table S2). No significant differences were observed in any species related to sex (Supplementary Table S3). 

## 4. Discussion

Both in dogs and cats, anemic patients showed a higher IRF compared to non-anemic animals, independently of the presence of reticulocytosis. Traditionally, the increase in reticulocyte numbers has been seen as a quantitative measure of the effectiveness of erythropoiesis, while the elevation in the IRF is considered an index of the acceleration of erythropoiesis [7,14,15]. An accelerated erythropoiesis indicates the liberation to the bloodstream of markedly young and immature reticulocytes and is a closer reflection of the intensity of erythropoietic stimulation [15]. This response is thought to be partially mediated by erythropoietin [6,16]. It is noteworthy that some patients can show an accelerated erythropoiesis without accompanying reticulocytosis due to bone marrow failure or an incompetence to efficiently elevate the total number of circulating reticulocytes [7]. 

Our findings demonstrate that hemolysis and hemorrhage cause an accelerated and effective erythropoiesis, with both reticulocytosis and a left-shift in the reticulocyte distribution to younger cells with higher fluorescence. Notably, dogs and cats with NRA also showed a certain degree of an accelerated (although ineffective) erythropoietic response (elevation in immature subsets of reticulocytes without reticulocytosis). 

In this study, we found significant differences in the IRF between dogs with NRA and RA, as previously reported [9]. Moreover, we also observed differences in other subpopulations such as HFR, MFR, and LFR, demonstrating that every aspect of reticulocyte dynamics is markedly different between both syndromes. 

In human medicine, the IRF is used to detect the recovery of bone marrow activity after chemotherapy and predict the success of bone marrow transplants [17,18]. In anemic patients, the IRF is an early indicator of bone marrow activity, preceding the appearance of reticulocytosis [19]. Thus, high IRF values can be used to differentiate PRA from NRA. Similar to a previous report [3], we found a higher IRF in PRA compared to both BMF and OTH in dogs. Remarkably, the IRF values in dogs with PRA were similar to those observed in patients with HEM (even though the former did not show reticulocytosis at presentation). However, when we tried to establish an IRF cut-off value to differentiate between PRA and other NRA, both sensitivity and specificity were worse than previously reported [3]. While this could be attributed to differences in the analyzer or classification of patients, it should be mentioned that the number of animals included in that previous study was lower than ours (25 vs. 137). 

Concerning cats, PRA also showed a significantly higher IRF compared to other causes of NRA. In this species, the proposed cut-off value demonstrated higher sensitivity compared to dogs, although specificity was moderate. Regardless of these shortcomings, our findings indicate that the IRF can be considered a valuable tool for the early identification of dogs and cats with PRA.

While the IRF is also used in human medicine to differentiate between causes of NRA (detect latent iron deficiency in blood donors, predict the appearance of bone marrow aplasia, etc.) [6,20], no publications in veterinary medicine are available in this regard. Here, we demonstrate that dogs and cats with BMF show a lower IRF compared to dogs with OTH. This finding contrasts reports in human medicine, where patients with aplastic anemia, myeloid leukemia, or myelodysplastic syndromes usually show a high IRF compared to those with chronic renal failure or iron deficiency anemia [7,21]. Whether this difference is due to species-specific idiosyncrasies or linked to dissimilar causes behind each subgroup (i.e., nutritional iron deficiency being a common cause of anemia in humans) should be investigated. 

This is the first study evaluating the IRF in different causes of RA in veterinary medicine. While no differences were found in any erythrocytic parameter or reticulocyte counts between animals with HEM and LYS, reticulocyte subpopulations were significantly dissimilar between both groups in both species. Moreover, the cut-off values for the IRF could be established with good to excellent accuracy for differentiating these two syndromes. Previous studies in human medicine have proven that hemolytic diseases are characterized by a markedly high IRF, indicating a pronounced compensatory bone marrow hyperplasia [22,23]. Moreover, hemolytic anemias usually cause a more robust bone marrow hyperplasia than hemorrhagic ones [2], mostly due to maintained iron availability and a possible effect of hemolysis-induced inflammation on the hematopoietic demand [24]. Our findings concur with these conclusions and underline the utility of the IRF for the differential diagnosis of these two common hematologic problems. Nonetheless, clinicians should be cautious in interpreting the IRF alone in these patients due to the possibility of overlapping, and it would always be advisable to consider the additional information from other diagnostic tests, clinical history, and signs. 

Cats are commonly characterized by lower reticulocyte counts compared to dogs, even when facing RA [25]. Nonetheless, healthy cats showed a higher IRF compared to dogs, which also can be seen when comparing any subgroup of NRA or RA between these species. Whether this high IRF is related to a species-specific idiosyncrasy in the maturation or liberation of reticulocytes or to differences in the fluorescence of these cells should be further investigated. 

Due to variations in the type of dye and algorithms used to identify these subpopulations, reference intervals (RIs) are usually not interchangeable between different automated analyzers [5]. Previous ranges published in healthy dogs using the Sysmex XT-2000iV analyzer found similar values for every reticulocyte subpopulation compared to our results [26,27]. On the other hand, a previous canine study using the ADVIA120 analyzer demonstrated lower HFR and IRF percentages compared to our results [28]. In cats, a previous study using the Sysmex XT-2000iV showed a lower IRF, HFR, and MFR compared to our data [29]. While differences in algorithms or staining protocols could explain these divergences, it should also be considered that our study included a larger population of dogs and cats compared to previous ones. 

Correlations between the IRF and other erythrocytic parameters have been inconsistently found in human medicine [7]. It is noteworthy that the IRF was not strongly correlated with the RET, neither in healthy nor in anemic animals, which emphasizes that accelerated and effective erythropoiesis can be independent from each other. 

No differences in reticulocyte subpopulations were found depending on sex in our study, similar to previously published data on dogs and cats [29,30]. On the other hand, young cats did show a lower IRF compared to older ones. While specific pediatric RIs have been established in humans, showing lower values compared to adults [31], age lacked any effect on these parameters in a previous study in cats [29]. In this last study, body weight did influence the values of the IRF, LFR, and MFR. Since we did not collect weight data from our population, we cannot determinate whether this age effect observed in cats is secondary to this effect, and further studies using more cats or groups with narrow ages are compelling.

In human medicine, the IRF is not only used for diagnosis, but also in the treatment monitoring of several types of anemia [20]. For example, the IRF can guide mineral replacement (cobalamin, folates, and iron) in the treatment of nutritional anemias [7] or the use of erythropoiesis-stimulating agents in anemia due to renal failure, AIDS, or myelodysplastic syndromes [5]. Whether these interesting clinical applications can be extrapolated to veterinary medicine is still to be studied. 

The present study has some limitations that warrant further investigation. First, the anemia in some patients could have been multifactorial, with several causative factors. Second, since serial CBCs were not available for every patient, some patients with PRA could have been classified as OTH. While we discarded any animal with an unclear classification, we cannot categorically dismiss these shortcomings. Third, some animals were excluded from our study due to a failed follow-up or incomplete diagnostic workup. Finally, we have focused our findings on the IRF. Although HFR is a more specific indicator of highly immature reticulocytes in circulation, it usually shows a higher coefficient of variation and is seldom used in human medicine [8]. Nonetheless, a previous preliminary study in our center did observe similar findings between subgroups using HFR [32].

## 5. Conclusions

This study demonstrates that IRF can differentiate between causes of NRA and RA in both dogs and cats using the Sysmex XN-1000V analyzer. Moreover, we have established cut-off values for an accurate differentiation between hemolytic and hemorrhagic anemias in both species. The RIs for reticulocyte subpopulations in the Sysmex XN-1000V were also reported in both species. 

Thus, the measurement of the IRF in this analyzer can help clinicians to further classify the type of anemia in both species, being a rapid and reliable parameter for the evaluation of these patients. Further research on new applications of IRF in veterinary medicine (treatment monitoring) is compelling. 

## Figures and Tables

**Table 1:** Reticulocyte and erythrocytic parameters in dogs and cats on the Sysmex XN-1000V.

	Dogs			Cats		
	Healthy (*n* = 2654)	NRA (*n* = 603)	RA (*n* = 223)	Healthy (*n* = 393)	NRA (*n* = 148)	RA (*n* = 48)
HCT (%)	45.7 (9)	28.5 (10) ^1^	23.3 (12) ^1,2^	33.8 ± 7	14.6 ± 6 ^a^	15.5 ± 5 ^a^
RBC (10^6^/µL)	6.4 (1.4)	3.9 (1.4) ^1^	3.1 (1.8) ^1^	7.4 (2.3)	3.1 (2.3) ^a^	3.4 (1.8) ^a^
Hb (g/dL)	15.7 (3.6)	9.0 (3.4) ^1^	7.3 (3.7) ^1^	11.3 (2.4)	5.2 ± 1.9 ^a^	5.2 ± 1.9 ^a^
MCV (fL)	70.9 (5.3)	72.1 (8.1) ^1^	75.7 (10) ^1,2^	50.7 (14)	46.4 (10) ^a^	50.7 (26) ^a,b^
MCH (pg)	24.5 (2.2)	23.7 (2.2) ^1^	24.2 (2.7) ^2^	14.8 (4.9)	16.1 (3.0) ^a^	17.4 (5.2) ^a^
MCHC (g/dL)	34.5 (2.1)	32.7 (3.4) ^1^	31.9 (3.0) ^1,2^	33.2 (2.7)	34.2 (3.9) ^a^	32.9 (3.6) ^b^
RDW (fL)	35.2 (4.8)	37.2 (8.2) ^1^	43.8 (13) ^1,2^	33.9 (8.3)	39.4 (15) ^a^	45.5 (19) ^a,b^
RET (10^3^/µL)	62.2 (57)	42.6 (45) ^1^	172 (124) ^1,2^	16.1 (20)	10.0 (15) ^a^	90.2 (141) ^a,b^
LFR (%)	77.3 (15)	74.6 (20) ^1^	52.8 (21) ^1,2^	64.3 ± 16	57.2 ± 19 ^a^	42.0 ± 18 ^a,b^
MFR (%)	10.1 (6.8)	10.0 (8.1)	14.7 (7.1) ^1,2^	11.3 ± 4.8	11.6 ± 6.4	12.3 ± 4.6
HFR (%)	12.3 (10)	13.8 (15) ^1^	29.7 (21) ^1,2^	24.3 ± 15	29.9 ± 17 ^a^	45.5 ± 18 ^a,b^
IRF (%)	22.7 (15)	25.4 (20) ^1^	47.2 (21) ^1,2^	35.6 ± 16	41.4 ± 18 ^a^	57.9 ± 18 ^a,b^

Data are expressed as median (IQR, interquartile range) or mean ± standard deviation (SD), according to distribution. Hb, hemoglobin concentration; HCT, hematocrit; HFR, high fluorescence reticulocytes; IRF, immature reticulocyte fraction; LFR, low fluorescence reticulocytes; MCH, mean corpuscular hemoglobin; MCHC, mean corpuscular hemoglobin concentration; MCV, mean corpuscular volume; MFR, medium fluorescence reticulocytes; NRA, non-regenerative anemia; RA, regenerative anemia; RBC, red blood cell count; RDW, red blood cell distribution width; RET, absolute reticulocyte count. ^1^ *p* < 0.001 vs. healthy dogs. ^2^ *p* < 0.001 vs. NRA dogs. ^a^ *p* < 0.001 vs. healthy cats. ^b^ *p* < 0.02 vs. NRA cats.

**Table 2:** Reticulocyte and erythrocytic parameters in different causes of canine and feline non-regenerative anemia on the Sysmex XN-1000V.

Parameter	Dogs with NRA (*n* = 603)			Cats with NRA (*n* = 148)		
	BMF (*n* = 83)	OTH (*n* = 383)	PRA (*n* = 137)	BMF (*n* = 34)	OTH (*n* = 79)	PRA (*n* = 35)
HCT (%)	24.4 (12) ^1,2^	28.4 (9)	30.2 (9)	16.2 ± 6	14.7 ± 5	14.4 ± 6
RBC (10^6^/µL)	3.6 (1.9)	3.9 (1.9)	3.9 (1.1)	3.8 (2.2)	3.2 (2.3)	3.6 (2.5)
Hb (g/dL)	7.9 (3.8) ^1,2^	8.9 (3.4)	9.7 (2.9)	5.6 ± 2.2	5.1 ± 1.9	5.0 ± 1.9
MCV (fL)	72.9 (9.8)	72.6 (7.8)	73.3 (9.6)	45.2 (6.5)	45.1 (10)	46.8 (10)
MCH (pg)	23.6 (2.6)	23.9 (2.2)	24.1 (1.9)	16.3 (2.7)	15.7 (4.7)	16.4 (3.5)
MCHC (g/dL)	32.5 (3.7)	32.7 (3.3)	32.9 (3.5)	34.4 (3.2)	34.3 (4.0)	33.9 (6.1)
RDW (fL)	38.9 (10)	37.3 (9.0)	36.3 (6.6)	40.4 (14)	38.6 (18)	40.5 (19)
RET (10^3^/µL)	23.3 (4) ^1,2^	43.7 (46)	50.9 (42)	10.8 (15)	9.9 (10)	12.9 (20)
LFR (%)	85.4 (21) ^1,2^	74.0 (19) ^2,3^	70.9 (20) ^1,3^	74.6 ± 17 ^a,b^	57.4 ± 12 ^b,c^	45.5 ± 19 ^a,c^
MFR (%)	7.0 (8.5) ^1,2^	10.0 (7.8)	11.6 (7.8)	8.0 ± 6.1 ^a,b^	14.0 ± 6.4	11.6 ± 4.1
HFR (%)	6.5 (12) ^1,2^	14.3 (14)	16.3 (17)	17.4 ± 17 ^a,b^	28.5 ± 12 ^b,c^	42.9 ± 19 ^a,c^
IRF (%)	14.6 (21) ^1,2^	26.0 (19) ^2,3^	29.1 (20) ^1,3^	25.9 ± 18 ^a,b^	42.5 ± 12 ^b,c^	54.5 ± 19 ^a,c^

Data are expressed as median (IQR, interquartile range) or mean ± standard deviation (SD), according to distribution. BMF, bone marrow failure; Hb, hemoglobin concentration; HCT, hematocrit; HFR, high fluorescence reticulocytes; IRF, immature reticulocyte fraction; LFR, low fluorescence reticulocytes; MCH, mean corpuscular hemoglobin; MCHC, mean corpuscular hemoglobin concentration; MCV, mean corpuscular volume; MFR, medium fluorescence reticulocytes; NRA, non-regenerative anemia; OTH, other non-regenerative anemias; PRA, pre-regenerative anemia; RBC, red blood cell count; RDW, red blood cell distribution width; RET, absolute reticulocyte count. ^1^ *p* < 0.05 vs. OTH in dogs. ^2^ *p* < 0.05 vs. PRA in dogs. ^3^ *p* < 0.05 vs. BMF in dogs. ^a^ *p* < 0.05 vs. OTH in cats. ^b^ *p* < 0.05 vs. PRA in cats. ^c^ *p* < 0.05 vs. BMF in cats.

**Table 3:** Reticulocyte and erythrocytic parameters in different causes of canine and feline regenerative anemia on the Sysmex XN-1000V.

Parameter	Dogs with RA (*n* = 223)		Cats with RA (*n* = 48)	
	HEM (*n* = 129)	LYS (*n* = 94)	HEM (*n* = 20)	LYS (*n* = 28)
HCT (%)	23.3 (12)	23.3 (14)	18.2 ± 5	13.7 ± 5
RBC (10^6^/µL)	3.1 (1.8)	3.1 (1.9)	3.5 ± 1.1	2.8 ± 1.2
Hb (g/dL)	7.2 (3.7)	7.4 (4.0)	5.9 ± 1.7	4.8 ± 1.9
MCV (fL)	75.3 (10)	76.8 (12)	53.5 ± 9	51.9 ± 10
MCH (pg)	23.9 (2.9)	24.4 (3.1)	17.5 ± 3.0	18.3 ± 4.4
MCHC (g/dL)	31.5 (3.0)	32.2 (2.8)	32.7 ± 1.2	35.3 ± 5.7
RDW (fL)	43.8 (12)	43.8 (13)	47.6 ± 14	49.4 ± 15
RET (10^3^/µL)	165 (105)	177 (134)	142 ± 115	187 ± 335
LFR (%)	63.0 (23)	45.8 (11) ^1^	58.8 ± 14	30.1 ± 11 ^a^
MFR (%)	14.2 (8.2)	15.7 (6.0)	11.7 ± 4.2	12.9 ± 4.9
HFR (%)	20.4 (19)	38.7 (12) ^1^	29.4 ± 11	57.0 ± 14 ^a^
IRF (%)	37.0 (23)	54.2 (11) ^1^	41.1 ± 14	69.9 ± 11 ^a^

Data are expressed as median (IQR, interquartile range) or mean ± standard deviation (SD), according to distribution. Hb, hemoglobin concentration; HEM, hemorrhagic anemia; HCT, hematocrit; HFR, high fluorescence reticulocytes; IRF, immature reticulocyte fraction; LFR, low fluorescence reticulocytes; LYS, hemolytic anemia; MCH, mean corpuscular hemoglobin; MCHC, mean corpuscular hemoglobin concentration; MCV, mean corpuscular volume; MFR, medium fluorescence reticulocytes; RA, regenerative anemia; RBC, red blood cell count; RDW, red blood cell distribution width; RET, absolute reticulocyte count. ^1^ *p* < 0.05 vs. dogs with HEM. ^a^ *p* < 0.05 vs. cats with HEM.

**Table 4:** Cut-off values of IRF by receiver operating characteristic (ROC) curve analysis for the diagnosis of different causes of anemia in dogs and cats on the Sysmex XN-1000V.

		Cut-Off	Sensitivity (%)	Specificity (%)	AUC	*p* Value
Dogs	HEM vs. LYS	44.7	87.2	71.3	0.834	<0.0001
	BMF vs. other NRA	16.2	79.6	55.4	0.722	<0.0001
	OTH vs. other NRA	11.7	22.2	87.9	0.518	0.45
	PRA vs. other NRA	21.9	46.7	77.3	0.626	<0.0001
Cats	HEM vs. LYS	54.3	96.4	80.0	0.936	<0.0001
	BMF vs. other NRA	27.7	85.9	76.4	0.836	<0.0001
	OTH vs. other NRA	32.2	52.1	82.2	0.549	0.29
	PRA vs. other NRA	53	84.9	68.5	0.761	<0.0001

AUC, area under the curve; BMF, bone marrow failure; HEM, hemorrhagic anemia; LYS, hemolytic anemia; NRA, non-regenerative anemias; OTH, other non-regenerative anemias; PRA, pre-regenerative anemia.

**Table 5:** Reference intervals for reticulocyte subpopulations in dogs and cats on the Sysmex XN-1000V.

	Healthy Dogs (*n* = 2654)	Healthy Cats (*n* = 393)
LFR (%)	77.3 (56.0–93.2)	66.7 (31.5–91.8)
MFR (%)	10.1 (2.3–19.6)	11. 4 (1.0–21.1)
HFR (%)	12.3 (2.5–29.8)	20.7 (3.4–59.1)
IRF (%)	22.7 (6.8–44.0)	33.3 (8.2–68.5)

HFR, high fluorescence reticulocytes; IRF, immature reticulocyte fraction; LFR, low fluorescence reticulocytes; MFR, medium fluorescence reticulocytes.

## Data Availability

The data presented in this study are available upon request from the corresponding author. The data are not publicly available due to privacy laws concerning the animals involved.

## Supplementary Materials

The following supporting information can be downloaded at: https://www.mdpi.com/article/10.3390/ani14020349/s1, Figure S1: Frequency distributions and reference intervals for IRF in healthy dogs; Figure S2: Frequency distributions and reference intervals for IRF in healthy cats; Table S1: Correlations between erythrocytic and reticulocyte parameters in anemic dogs (A) and cats (B); Table S2: Reticulocyte parameters in healthy dogs (A) and cats (B) grouped by age; Table S3: Reticulocyte parameters in dogs (A) dogs and cats (B) grouped by sex.

## References

[1] Comazzi S., Pieralisi C., Bertazzolo W. (2004). Haematological and biochemical abnormalities in canine blood: Frequency and associations in 1022 samples. J. Small Anim. Pract..

[2] Tvedten H., Brooks M.B., Harr K.E., Seelig D.V., Wardrop K.J., Weiss D.J. (2022). Classification and laboratory evaluation of anemia. Schalm’s Veterinary Hematology.

[3] Jung J.H., Yang Y., Seo D., Cho S., Choi G., Kim Y. (2023). Clinical utility of immature reticulocyte fraction for identifying early red blood cell regeneration in anemic dogs. J. Vet. Intern. Med..

[4] Brugnara C. (2000). Reticulocyte cellular indices: A new approach in the diagnosis of anemias and monitoring of erythropoietic function. Crit. Rev. Clin. Lab. Sci..

[5] Piva E., Brugnara C., Spolaore F., Plebani M. (2015). Clinical utility of reticulocyte parameters. Clin. Lab. Med..

[6] Suria N., Kaur R., Mittal K., Palta A., Sood T., Kaur P., Kaur G. (2022). Utility of reticulocyte haemoglobin content and immature reticulocyte fraction in early diagnosis of latent iron deficiency in whole blood donors. Vox Sang..

[7] Buttarello M. (2016). Laboratory diagnosis of anemia: Are the old and new red cell parameters useful in classification and treatment, how?. Int. J. Lab. Hematol..

[8] Chang C.C., Kass L. (1997). Clinical significance of immature reticulocyte fraction determined by automated reticulocyte counting. Am. J. Clin. Pathol..

[9] Melendez-Lazo A., Tvarijonaviciute A., Ceron J.J., Planellas M., Pastor J. (2015). Evaluation of the relationship between selected reticulocyte parameters and inflammation determined by plasma C-reactive protein. J. Comp. Pathol..

[10] Rørtveit R., Saevik B.K., Eggertsdóttir A.V., Skancke E., Lingaas F., Thoresen S.I., Jansen J.H. (2015). Age-related changes in hematologic and serum biochemical variables in dogs aged 16–60 days. Vet. Clin. Pathol..

[11] Zaldivar-Lopez S., Marin L.M., Iazbik M.C., Westendorf-Stingle N., Hensley S., Couto C.G. (2011). Clinical pathology of Greyhounds and other sighthounds. Vet. Clin. Pathol..

[12] Safari S., Baratloo A., Elfil M., Negida A. (2016). Evidence based emergency medicine; Part 5 Receiver operation curve and area under the curve. Emergency.

[13] Friedrichs K.R., Harr K.E., Freeman K.P., Szladovits B., Walton R.M., Barnhart K.F., Blanco-Chavez J., American Society for Veterinary Clinical Pathology (2012). ASVCP reference interval guidelines: Determination of de novo reference intervals in veterinary species and other related topics. Vet. Clin. Pathol..

[14] Buttarello M., Bulian P., Farina G., Temporin V., Toffolo L. (2000). Frazione di reticolociti immaturi (IRF) e conteggio reticolocitario assoluto: Analisi multivariata e studio della covarianza in diversi subsets eritropoietici. Riv. Med. Lab. (JLM).

[15] D’Onofrio G., Kuse R., Foures C., Jour J.M., Pradella M., Zini G. (1996). Reticulocytes in haematological disorders. Clin. Lab. Haematol..

[16] Davis B.H., Ornvold K., Bigelow N.C. (1995). Flow cytometric reticulocyte maturity index: A useful laboratory parameter of erythropoietic activity in anemia. Cytometry.

[17] Luczynski W., Ratomski K., Wysocka J., Krawczuk-Rybak M., Jankiewicz P. (2006). Immature reticulocyte fraction (IRF)—An uni-versal marker of hemopoiesis in children with cancer?. Adv. Med. Sci..

[18] Grazziutti M.L., Dong L., Miceli M.H., Cottler-Fox M., Krishna S.G., Fassas A., van Rhee F., Barlogie B.M., Anaissie E.J. (2006). Recovery from neutropenia can be predicted by the immature reticulocyte fraction several days before neutrophil recovery in autologous stem cell transplant recipients. Bone Marrow Transplant..

[19] Morkis I.V., Farias M.G., Rigoni L.D., Scotti L., Gregianin L.J., Daudt L.E., da R Silla L.M., Paz A.A. (2015). Assessment of immature platelet fraction and immature reticulocyte fraction as predictors of engraftment after hematopoietic stem cell transplantation. Int. J. Lab. Hematol..

[20] Moloney C., Stavroulaki E.M., Augusto M. (2023). Reference intervals for reticulocyte indices, immature reticulocyte fraction, and the percentage of hypochromic red blood cells in adult large breed dogs using the ADVIA 2120 hematology analyzer. Vet. Clin. Pathol..

[21] Watanabe K., Kawai Y., Takeuchi K., Shimizu N., Iri H., Ikeda Y., Houwen B. (1994). Reticulocyte maturity as an indicator for estimating qualitative abnormality of erythropoiesis. J. Clin. Pathol..

[22] Bobee V., Daliphard S., Schrapp A., Lahary A. (2018). Screening of hereditary spherocytosis and pyruvate kinase deficiency by automated blood count using erythrocytic and reticulocytic parameters. Int. J. Lab. Hematol..

[23] Mullier F., Lainey E., Fenneteau O., Da Costa L., Schillinger F., Bailly N., Cornet Y., Chatelain C., Dogne J.M., Chatelain B. (2011). Additional erythrocytic and reticulocytic parameters helpful for diagnosis of hereditary spherocytosis: Results of a multicentre study. Ann. Hematol..

[24] Moreau R., Malu D.T., Dumais M., Dalko E., Gaudreault V., Romero H., Martineau C., Kevorkova O., Sanchez-Dardon J., Dodd E.L. (2012). Alterations in bone and erythropoiesis in hemolytic anemia: Comparative study in bled, phenylhydrazine-treated and Plasmodium-infected mice. PLoS ONE.

[25] Schaefer D.M.W., Brooks M.B., Harr K.E., Seelig D.V., Wardrop K.J., Weiss D.J. (2022). Hematology of cats. Schalm’s Veterinary Hematology.

[26] Mathers A., Evans G.O., Bleby J. (2012). Reticulocyte measurements in rat, dog and mouse whole blood samples using the Sysmex XT-2000iV. Comp. Clin. Pathol..

[27] Serra M., Freeman K.P., Campora C., Sacchini F. (2012). Establishment of canine hematology reference intervals for the Sysmex XT-2000iV hematology analyzer using a blood donor database. Vet. Clin. Pathol..

[28] Moritz A., Fickenscher Y., Meyer K., Failing L., Weiss D.J. (2004). Canine and feline hematology reference values for the ADVIA 120 hematology system. Vet. Clin. Pathol..

[29] Granat F., Geffre A., Bourges-Abella N., Mortier J., Theron M., Fauchon E., Braun J., Trumel C. (2014). Feline reference intervals for the Sysmex XT-2000iV and the ProCyte DX haematology analysers in EDTA and CTAD blood specimens. J. Feline Med. Surg..

[30] Bourges-Abella N., Geffre A., Concordet D., Braun J.P., Trumel C. (2011). Canine reference intervals for the Sysmex XT-2000iV hematology analyzer. Vet. Clin. Pathol..

[31] Bracho F.J. (2022). Reference intervals of automated reticulocyte count and immature reticulocyte fraction in a pediatric population. Int. J. Lab. Hematol..

[32] Perez-Ecija A., Mendoza F.J. SEED Veterinary Hematology: Diagnostic Use of the Reticulocyte Maturity Indices Provided by the Sysmex XN-V Analyser. https://www.sysmex.co.uk/education/library/documents/detail/seed-diagnostic-use-of-the-reticulocyte-maturity-indices-on-xn-v.html.

