# Supplementary material for: The Immature Reticulocyte Fraction (IRF) in the Sysmex XN-1000V Analyzer Can Differentiate between Causes of Regenerative and Non-Regenerative Anemia in Dogs and Cats
Source: Animals (Basel). 2024 Jan 22;14(2):349. doi: 10.3390/ani14020349 (PMC10812539; doi:10.3390/ani14020349)
Supplement: Supplementary file 1 [file animals-14-00349-s001.zip › Supplementary Figure.pdf]

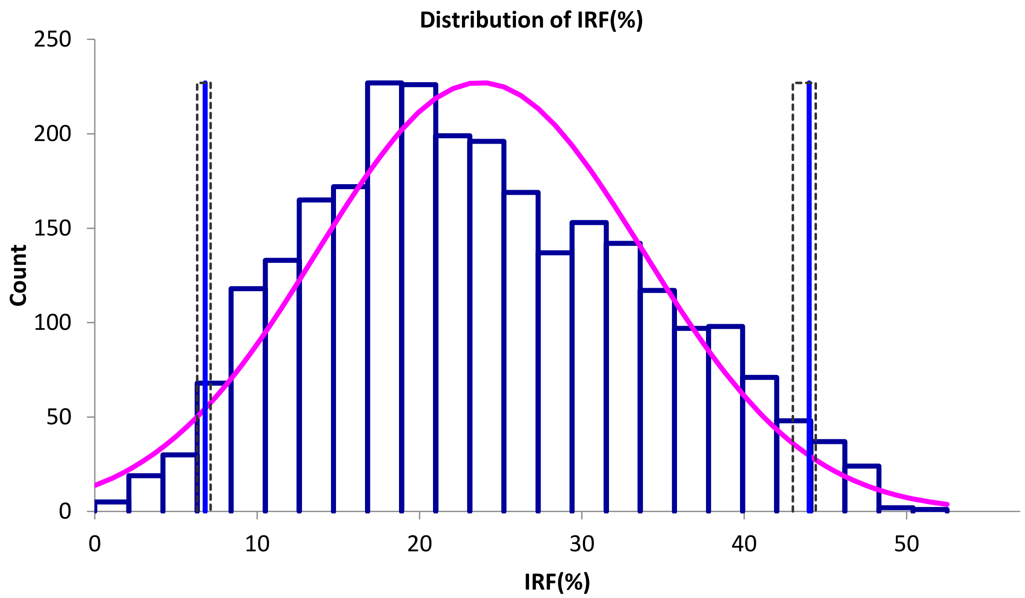

Supplementary Figure S1. Frequency distributions and reference intervals for IRF in healthy dogs. The observed distribution is represented by the vertical black columns, while the red curve is the fitted distribution. Reference limits are drawn as vertical blue lines. Dotted bar surrounding those limits are the 90% confidence intervals.

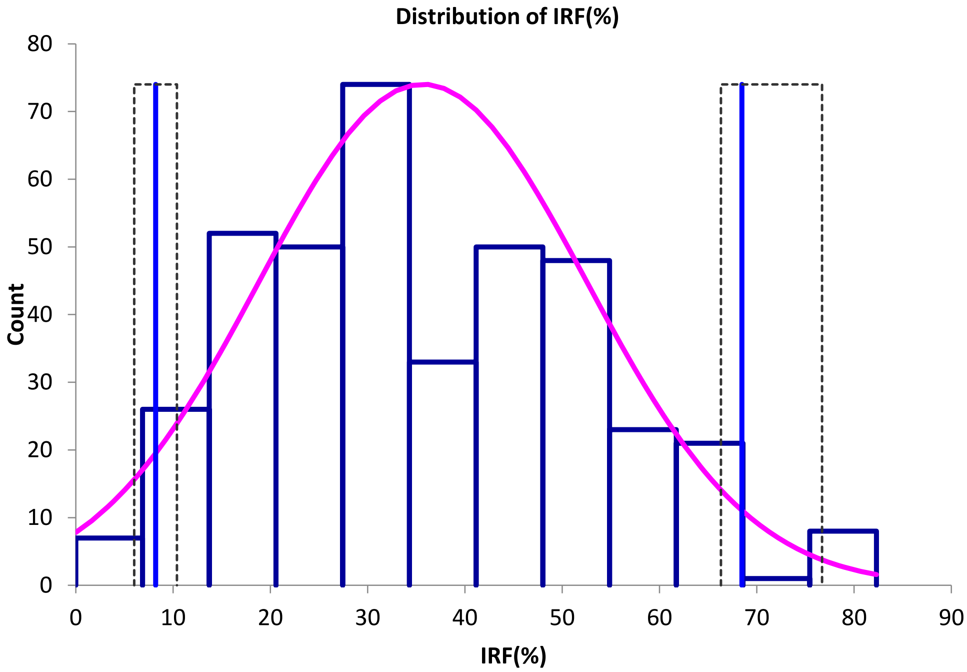

Supplementary Figure S2. Frequency distributions and reference intervals for IRF in healthy cats. The observed distribution is represented by the vertical black columns, while the red curve is the fitted distribution. Reference limits are drawn as vertical blue lines. Dotted bar surrounding those limits are the 90% confidence intervals.
