# Supplementary material for: The Immature Reticulocyte Fraction (IRF) in the Sysmex XN-1000V Analyzer Can Differentiate between Causes of Regenerative and Non-Regenerative Anemia in Dogs and Cats
Source: Animals (Basel). 2024 Jan 22;14(2):349. doi: 10.3390/ani14020349 (PMC10812539; doi:10.3390/ani14020349)
Supplement: Supplementary file 1 [file animals-14-00349-s001.zip › Supplementary Table.pdf]

Supplementary Table S1. Correlations between erythrocytic and reticulocyte parameters in anemic dogs (A) and anemic cats (B).

(A)

	**RBC (10^6/µL)**	**Hb (g/dL)**	**HCT (%)**	**MCV (fL)**	**MCH (pg)**	**MCHC (g/dL)**	**RDW (fL)**	**RET (10^6/µL)**	**LFR (%)**	**MFR (%)**	**HFR (%)**	**IRF (%)**
LFR (%)	0.25	0.20	0.19	- 0.19	- 0.11	0.09	- 0.27	- 0.54	1.00	- 0.68	- 0.95	- 1.00
MFR (%)	0.00	0.02	0.00	- 0.01	0.01	0.04	0.17	0.48	- 0.68	1.00	0.45	0.68
HFR (%)	- 0.28	- 0.23	- 0.21	0.23	0.14	- 0.13	0.26	0.47	- 0.95	0.45	1.00	0.95
IRF (%)	- 0.25	- 0.20	- 0.19	0.187	0.12	-0.09	0.27	0.54	- 1.00	0.68	0.95	1.00

Data are expressed as Spearman's rank correlation coefficient (ϱ). Hb, hemoglobin concentration; HCT, hematocrit; HFR, high fluorescence reticulocytes; IRF, immature reticulocyte fraction; LFR, low fluorescence reticulocytes; MCH, mean corpuscular hemoglobin; MCHC, mean corpuscular hemoglobin concentration; MCV, mean corpuscular volume; MFR, medium fluorescence reticulocytes; RBC, red blood cell count; RDW, red blood cell distribution width; RET, absolute reticulocyte count.

(B)

	**RBC (10^6/µL)**	**Hb (g/dL)**	**HCT (%)**	**MCV (fL)**	**MCH (pg)**	**MCHC (g/dL)**	**RDW (fL)**	**RET (10^6/µL)**	**LFR (%)**	**MFR (%)**	**HFR (%)**	**IRF (%)**
LFR (%)	0.21	0.26	0.24	- 0.09	- 0.08	0.01	- 0.08	- 0.21	1.00	-0.27	- 0.95	- 1.00
MFR (%)	- 0.19	- 0.18	- 0.17	0.10	0.07	0.01	0.15	0.01	- 0.27	1.00	- 0.03	0.27
HFR (%)	- 0.16	- 0.21	- 0.20	0.07	0.06	- 0.01	0.04	0.22	- 0.95	-0.03	1.00	0.95
IRF (%)	- 0.21	- 0.26	- 0.24	0.09	0.08	- 0.01	0.08	0.21	- 1.00	0.27	0.95	1.00

Data are expressed as Pearson correlation coefficient (r). Hb, hemoglobin concentration; HCT, hematocrit; HFR, high fluorescence reticulocytes; IRF, immature reticulocyte fraction; LFR, low fluorescence reticulocytes; MCH, mean corpuscular hemoglobin; MCHC, mean corpuscular hemoglobin concentration; MCV, mean corpuscular volume; MFR, medium fluorescence reticulocytes; RBC, red blood cell count; RDW, red blood cell distribution width; RET, absolute reticulocyte count.

Supplementary Table S2. Reticulocyte parameters in healthy (a) dogs and (b) cats grouped by age.

(A)

Parameter	Healthy dogs		
	**Group 1: < 1 yo (n= 297)**	**Group 2: 1-10 yo (n = 1625)**	**Group 3: > 10 yo (n = 732)**
RET ($10^3/\mu L$)	62.6 (60)	64.3 (55)	58.1 (57)
LFR (%)	77.9 (14)	77.1 (16)	77.6 (15)
MFR (%)	11.0 (7.9)	10.1 (6.9)	9.7 (6.4)
HFR (%)	11.9 (10)	12.4 (11)	12.2 (10)
IRF (%)	22.1 (16)	22.9 (14)	22.4 (15)

Data are expressed as median (IQR, interquartile range). HFR, high fluorescence reticulocytes; IRF, immature reticulocyte fraction; LFR, low fluorescence reticulocytes; MFR, medium fluorescence reticulocytes; RET, absolute reticulocyte count.

(B)

Parameter	Healthy cats		
	**Group 1: < 1 yo (n= 31)**	**Group 2: 1-10 yo (n = 168)**	**Group 3: > 10 yo (n = 194)**
RET ($10^3/\mu L$)	15.4 (16)	18.7 (24)	15.5 (17)
LFR (%)	77.1 ± 13	64.1 ± 16[1]	62.4 ± 17[1]
MFR (%)	9.4 ± 4.9	12.1 ± 4.9[1]	10.9 ± 4.7
HFR (%)	11.3 (10)	21.2 (11)[1]	22.1 (24)[1]
IRF (%)	22.8 ± 13	35.8 ± 16[1]	37.6 ± 17[1]

Data are expressed as median (IQR, interquartile range) or mean ± standard deviation (SD), according to distribution. HFR, high fluorescence reticulocytes; IRF, immature reticulocyte fraction; LFR, low fluorescence reticulocytes; MFR, medium fluorescence reticulocytes; RET, absolute reticulocyte count. [1] $p < 0.05$ vs. Group 1.

Supplementary Table S3. Reticulocyte parameters in (a) dogs and (b) cats grouped by sex.

(A)

Parameter	Healthy dogs	
	**Female (n= 1443)**	**Male (n = 1211)**
RET ($10^3/\mu L$)	65.4 (57)	58.3 (57)
LFR (%)	76.7 (15)	78.0 (15)
MFR (%)	10.4 (6.6)	9.7 (7.2)
HFR (%)	12.6 (10)	11.8 (11)
IRF (%)	23.3 (15)	22.0 (15)

Data are expressed as median (IQR, interquartile range). HFR, high fluorescence reticulocytes; IRF, immature reticulocyte fraction; LFR, low fluorescence reticulocytes; MFR, medium fluorescence reticulocytes; RET, absolute reticulocyte count.

(B)

Parameter	Healthy cats	
	**Female (n= 229)**	**Male (n = 164)**
RET ($10^3/\mu L$)	15.2 (17)	18.6 (25)
LFR (%)	66.7 (27)	66.8 (26)
MFR (%)	11.2 ± 4.9	11.4 ± 4.7
HFR (%)	20.6 (22)	21.3 (21)
IRF (%)	33.3 (27)	33.1 (26)

Data are expressed as median (IQR, interquartile range) or mean ± standard deviation (SD), according to distribution. HFR, high fluorescence reticulocytes; IRF, immature reticulocyte fraction; LFR, low fluorescence reticulocytes; MFR, medium fluorescence reticulocytes; RET, absolute reticulocyte count.

(B)

Parameter	Healthy cats		
	**Group 1: < 5 yo (n= 77)**	**Group 2: 5-10 yo (n = 122)**	**Group 3: > 10 yo (n = 194)**
ARC (10^3/μL)	17.1 (22)	17.7 (24)	15.5 (17)
LFR (%)	69.1 ± 14	64.3 ± 17	62.4 ± 17[1]
MFR (%)	11.6 ± 5.2	11.7 ± 6.0	10.9 ± 4.7
HFR (%)	16.7 (16)	20.7 (22)	22.1 (24)[1]
IRF (%)	30.8 ± 14	35.7 ± 17	37.6 ± 17[1]

Data are expressed as median (IQR, interquartile range) or mean ± standard deviation (SD), according to distribution. ARC, absolute reticulocyte count; HFR, high fluorescence reticulocytes; IRF, immature reticulocyte fraction; LFR, low fluorescence reticulocytes; MFR, medium fluorescence reticulocytes. [1] $p < 0.05$ vs. Group 1.
